# A randomized phase II study of full dose gemcitabine versus reduced dose gemcitabine and nab-paclitaxel in vulnerable patients with non-resectable pancreatic cancer (DPCG-01)

**DOI:** 10.1186/s12885-023-11035-6

**Published:** 2023-06-16

**Authors:** Louise Skau Rasmussen, Stine B. Winther, Inna M. Chen, Britta Weber, Lise Ventzel, Gabor Liposits, Julia Sidenius Johansen, Sönke Detlefsen, Ida Egendal, Susy Shim, Signe Christensen, Per Pfeiffer, Morten Ladekarl

**Affiliations:** 1 Department of Oncology and Clinical Cancer Research Center, Aalborg University Hospital, Aalborg, Denmark; 2 Department of Oncology, Odense University Hospital, Odense, Denmark; 3 Department of Oncology, Herlev-Gentofte University Hospital, Copenhagen, Denmark; 4 Department of Oncology, Aarhus University Hospital, Aarhus, Denmark; 5 Department of Oncology, University Hospital of Southern Denmark, Vejle, Denmark; 6 Department of Oncology, Gødstrup Hospital, Herning, Denmark; 7 Department of Oncology, Copenhagen University Hospital – Herlev and Gentofte, Herlev, Denmark; 8 Department of Pathology, Odense University Hospital, and Department of Clinical Research, Faculty of Health Sciences, University of Southern Denmark, Odense, Denmark; 9 Center for Clinical Data Science (CLINDA), and Clinical Cancer Research Center, Aalborg University and, Aalborg University Hospital, Aalborg, Denmark; 10 Department of Oncology and Clinical Cancer Research Center, Aalborg University Hospital, and Department of Clinical Medicine, Aalborg University, Aalborg, Denmark; 11 Department of Oncology, Odense University Hospital, and Department of Clinical Research, Faculty of Health Sciences, University of Southern Denmark, Odense, Denmark

**Keywords:** Chemotherapy dose, Comorbidity, Frail, Older patients, Pancreatic cancer, Randomized study, Quality of life, Toxicity

## Abstract

**Background:**

According to current evidence, the best treatment for fit patients with non-resectable pancreatic cancer (PC) is combination chemotherapy, whereas frail patients are recommended gemcitabine (Gem) monotherapy. Randomized controlled trials in colorectal cancer and a post-hoc analysis of gemcitabine and nab-paclitaxel (GemNab) in PC suggest, however, that reduced dose of combination chemotherapy may be feasible and more efficient compared to monotherapy in frail patients. The aim of this study is to investigate whether reduced dose GemNab is superior to full dose Gem in patients with resectable PC, who are not candidates for full dose combination chemotherapy in first line.

**Methods:**

The Danish Pancreas Cancer Group (DPCG)-01 trial is a national multicenter prospective randomized phase II trial. A total of 100 patients in ECOG performance status 0–2 with non-resectable PC, not candidate for full dose combination chemotherapy in first line, but eligible for full dose Gem, will be included. Patients are randomized 1:1 to either full dose Gem or GemNab in 80% of recommended dose.

The primary endpoint is progression-free survival. Secondary endpoints are overall survival, overall response rate, quality of life, toxicity and rate of hospitalizations during treatment. The correlation between blood inflammatory markers, including YKL-40 and IL-6, circulating tumor DNA, and tissue biomarkers of resistance to chemotherapy and outcome will be explored. Finally, the study will include measures of frailty (G8, modified G8, and chair-stand-test) to assess whether scoring would enable a personalized allocation to different treatments or indicates a possibility for interventions.

**Discussion:**

Single-drug treatment with Gem has for frail patients with non-resectable PC been the main treatment option for more than thirty years, but the impact on outcome is modest. If improved results and sustained tolerability with reduced dose combination chemotherapy can be shown, this could change the future practice for this increasing group of patients.

**Trial registration:**

ClinicalTrials.gov Identifier: NCT05841420.

Secondary Identifying No: N-20210068.

EudraCT No: 2021–005067-52.

Protocol version: 1.5, 16-MAY-2023.

**Supplementary Information:**

The online version contains supplementary material available at 10.1186/s12885-023-11035-6.

## Background

The incidence of pancreatic cancer (PC) is increasing and PC is one of the most lethal diseases. In the United States, PC is currently ranked as the 4^th^ leading cause of cancer-related death and projected to become the 2^nd^ leading cause by 2040 [1, 2]. This is due to changing demographic characteristics along with a reduction in incidence of tobacco-related malignancies and improved prognosis of most other cancers [2]. The incidence of PC is highest among patients between 70–80 years [2], and older persons comprise the world’s fastest growing age group [3].

The majority of PC patients are diagnosed in a non-resectable stage; 30% in a locally advanced and 50% in a metastatic stage [4]. The treatment option for most of these patients is palliative chemotherapy, where the goal is to achieve an adequate balance between toxicity and efficacy. Unfortunately, only a few randomized studies of systemic treatments performed in PC have had impact on practice. Thirty-six years ago, Burris et al compared gemcitabine (Gem) with 5-fluorouracil (5-FU) in 126 patients with locally advanced and metastatic PC [5]. Gem resulted in a median overall survival (mOS) of 5.7 months, which was slightly superior to 5-FU. Moreover, more patients achieved clinical benefit when treated with Gem [5]. In the trial, 70% of Gem-treated patients had a reduced Karnofsky performance status (KPS) of 50–70 [5]. Since then, Gem has been a recommended treatment option for patients with non-resectable PC and Eastern Cooperative Oncology Group (ECOG) performance status (PS) ≤ 2 [6]. In 2011, the combination of 5-FU, leucovorin, irinotecan and oxaliplatin (FOLFIRINOX) showed an improvement in mOS in metastatic PC of 4.3 months compared to Gem [7]. However, grade 3–4 adverse events (AEs), such as febrile neutropenia, diarrhea, and sensory neuropathy, were frequent [7] and FOLFIRINOX is mainly preserved for younger and fit patients [6]. Two years later, Von Hoff et al showed that Gem plus nab-paclitaxel (GemNab) in patients with metastatic PC was superior to Gem, with a modest improvement in mOS of 1.8 months [8]. Most patients were in good PS. Grade 3–4 AEs with diarrhea and sensory neuropathy were more frequent in the GemNab treated group [8]. Thus, the European Society for Medical Oncology (ESMO) guideline recommends that GemNab should be offered primarily to patients with PS 0–1 and only to very selected patients in PS 2 in need for a response [9]. Finally, results of the combination of liposomal irinotecan, 5-FU, leucovorin and oxaliplatin (NALIRINOX) in fit patients with metastatic PC were recently presented in abstract form, showing an improvement in mOS of 1.9 months compared to GemNab [10].

Hampering the potential benefit of more intense treatment, patients with PC very often present with affected overall health status, either secondary to the disease or because of comorbidities [11]. PS is commonly used for the clinical assessment of a patient's ability to tolerate treatment, although PS does not consider age, comorbidities, or other aspects of frailty [12]. Patients with KPS of 50–70 or PS of 2 or worse have usually been excluded from randomized clinical trials (RCTs) investigating combination chemotherapy in PC [7, 8]. As a result, there is sparse evidence on efficacy and toxicity of combination chemotherapy relative to single agent treatment in poor PS patients. In the MPACT trial by Von Hoff et al, only 8% had a KPS of 70 [8]. In a *post-hoc* subgroup analysis, unconventionally including a majority of patients with KPS 80 in the poor PS population, an improved mOS [13] and similar dose reductions and frequencies of AEs were found in patients with KPS 70–80 treated with GemNab vs Gem compared to those with KPS 90–100 [14, 15]. In the entire cohort, mOS was surprisingly shorter for GemNab-treated patients without dose reduction compared to those with (6.9 vs 11.4 months), and for those who completed treatment without delay compared to those who had dose delay (6.2 vs 10.1 months). Dose reduction or delay in response to toxicity allowed patients to receive more cycles and higher cumulative dose [14]. Dose reduction of nab-paclitaxel at start of treatment was investigated in a phase II trial of 221 PC patients with PS 2 [16]. Patients were randomized to GemNab in standard dose or standard dose Gem plus reduced dose nab-paclitaxel at 100 mg/m^2^ (80%) [16]. No significant differences in median progression-free survival (mPFS) and grade 3–4 AEs were observed [16]. However, doses were often reduced resulting in relative dose intensities of around 75% in both arms [16].

Frailty increases with age due to increased prevalence of comorbidities, polypharmacy, and compromised organ function, and in the Danish population one fifth of patients treated with chemotherapy for PC are ≥ 75 years [17]. Despite this, the use of chemotherapy in the elderly has not been well investigated in RCTs, where median ages of included patients were only 61–66 years [7, 8, 18–21]. Older patients more frequently receive treatment with Gem compared to combination chemotherapy [17, 22, 23]. In a subgroup analysis of the MPACT trial of patients ≥ 65 years, only a non-significant trend for survival benefit of GemNab was found [13]. In a register-based cohort using multivariate analysis, patients treated with combination chemotherapy had better mOS than those treated with Gem only, regardless of age [17]. Reports on toxicity among elderly patients are divergent. In a RCT of Gem versus GemNab, tolerability and need for dose modification for patients < 65 and ≥ 65 years were similar [14]. However, a retrospective assessment of 116 patients with a median age of 77 years showed a higher frequency of severe AEs with GemNab than reported in RCTs [24]. Furthermore, fatigue and decreased appetite were more frequent in patients ≥ 70 years treated with GemNab compared to younger patients [25]. The impact of dose reduction in elderly patients was assessed in a retrospective study of 73 patients with a median age of 73 years treated with GemNab on day 1 and 15 in a 4-week cycle, showing low incidence of grade 3–4 AEs and rare dose reductions [26].

In conclusion, improvements in the prognosis of PC in the growing vulnerable population are highly needed and an approach to improve tolerability and efficacy of treatment could be dose reduction of combination chemotherapy [14, 16, 26]. The primary aim of this study is to evaluate whether reduced dose of GemNab is superior to standard dose Gem with respect to PFS in non-resectable PC patients not fit for full dose combination chemotherapy in first line.

## Design and methods

This study is a national multicenter prospective randomized phase II trial.

### Study endpoints

The primary endpoint is PFS. Secondary endpoints are OS, overall response rate (ORR), quality of life (QoL), toxicity and rate of hospitalizations during treatment. Exploratory endpoints include evaluation of pretreatment characteristics and geriatric screening tools as predictive markers, and correlation between plasma biomarkers of systemic inflammation, circulating tumor DNA (ctDNA), tissue biomarkers of resistance to chemotherapy, and outcome.

### Study population and eligibility criteria

Patients are recruited from six of seven oncological departments treating patients with PC in Denmark, covering 85% of the oncological PC population [27]. Approximately 350 patients yearly are treated with chemotherapy in first line in Denmark [28]. Patients will be assessed when they meet for their first consultation regarding first line palliative chemotherapy.

A full description of eligibility criteria is provided elsewhere (NCT05841420). Briefly, all patients included are at least 18 years of age with non-resectable, pathologically verified adenocarcinoma of the pancreas, not candidate for full dose combination chemotherapy but eligible for standard dose gemcitabine. Patients are in ECOG PS ≤ 2, with measurable or non-measurable disease, having adequate hematologic, liver and kidney function, and with toxicity of possible prior adjuvant chemotherapy resolved to National Cancer Institute (NCI) Common Terminology Criteria for Adverse Events (CTCAE) ver.5.0 < grade 2 [29]. Patients should provide oral and written informed consent and fertile patients must use adequate contraceptives.

Main exclusion criteria are eligibility for downstaging/preoperative chemotherapy, prior chemotherapy for PC except adjuvant therapy with recurrence occurring more than 6 months after end of treatment, and other conditions or therapy, which in the investigator’s opinion may pose a risk to the patient or interfere with the study objectives.

The intention-to-treat (ITT) population includes all randomized patients. The per protocol (PP) population includes all randomized patients who receive at least one dose of planned chemotherapy and will be the population for all safety analyses.

### Randomization and treatment

**Fig. 1:**
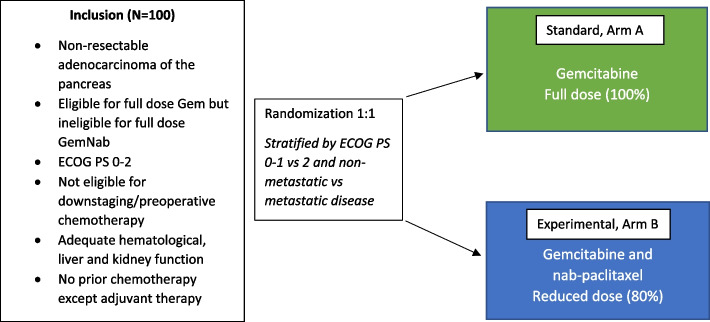
Summary of eligibility criteria and randomization

A total of 100 patients, 50 in each arm, will be included (Fig. 1). Patients will be randomized 1:1 to either Arm A or Arm B. Randomization will be stratified for ECOG PS (0–1 vs 2) and metastatic disease (present vs not present). In Arm A, patients will receive gemcitabine 1000 mg/m^2^ weekly on days 1, 8, and 15, every 4 weeks. Gemcitabine will be administered as an intravenous infusion for 30 min. In Arm B, patients are treated with gemcitabine 800 mg/m^2^ plus nab-paclitaxel 100 mg/m^2^ on day 1, 8 and 15, every 4 weeks. Both drugs will be administered as an intravenous infusion for 60 min. The doses of gemcitabine and nab-paclitaxel in arm B are similar to dose level -1 in the pivotal MPACT study protocol [8].

Patients may continue treatment until progressive disease (PD), unacceptable toxicity, withdrawal of consent, or until the treating physician judges that continued treatment poses an unacceptable risk to the patient’s health. In case of treatment discontinuation, patients are allowed further treatment according to Danish guidelines [6]. Patients will be followed-up every two months after treatment discontinuation to register further treatment, date of progression and vital status from the electronic health records.

### Dose modifications

For Arm A, two dose reductions are allowed, while only one dose reduction is allowed for Arm B (Table 1). Dose modifications, if needed, are done according to the MPACT study protocol, adapted to the Danish product summary [8, 30] as shown in supplementary Table S1-6. Dose-limiting neutropenia is generally managed by dose delay or reduction, however, the use of granulocyte-colony stimulating factor (G-CSF) is allowed in both arms. Dose-limiting thrombocytopenia is managed by dose delay or reduction. Non-hematological AEs CTC grade 3 or worse (excluding nausea/vomiting and alopecia) will lead to delay of treatment until resolved to AE CTC grade 1 or 0, and future dose will be reduced. For Arm B, peripheral neuropathy CTC grade 3 or worse will lead to delay of nab-paclitaxel until resolved to CTC grade 1 or 0, and future dose of nab-paclitaxel will be reduced. If patients are treated at the lowest dose level and experience dose limiting toxicity for more than three weeks, treatment is permanently aborted.

**Table 1:** Dose modifications for patients randomized to Arm A and B, respectively

	**Arm A**	**Arm B**	
**Dose level**	**Gemcitabine**	**Gemcitabine**	**Nab-paclitaxel**
0	1000 mg/m^2^	800 mg/m^2^	100 mg/m^2^
-1	800 mg/m^2^	600 mg/m^2^	75 mg/m^2^
-2	600 mg/m^2^	-	-

### Assessment plan

Study procedures are summarized in supplementary Table S7. Baseline assessments include a CT scan, demographics, medical history, ECOG PS, body surface area, and blood chemistry. Moreover, the European Organization for Research and Treatment of Cancer (EORTC) Quality of Life Questionnaire (QLQ)-C30 ver.3 is completed by the patient, and Charlson Comorbidity Index (CCI) score, Geriatric 8 (G8) score, modified Geriatric-8 (mG8) score, chair-stand-test and optional blood samples for biomarkers are done. Prior to each treatment course, hematology is evaluated. Prior to each chemotherapy cycle, toxicity, symptoms, hospitalizations, weight and ECOG PS are registered, and blood samples for biochemistry are taken.

Treatment effect is evaluated every 8 weeks by a CT-scan and serum cancer antigen (CA) 19–9. At the same time, blood samples, optional blood samples for biomarkers, a chair-stand-test, and the EORTC QLQ-C30 are performed.

### Assessment of primary and secondary endpoints

#### Progression-free survival

In the ITT population, PFS is defined as the time from date of randomization to the date of disease progression or date of death, whichever comes first. The date of PD is the date of scan, if progression is found on a CT scan, or date of visit, if progression is found clinically. PD at CT is defined according to Response Evaluation Criteria in Solid Tumours (RECIST) ver.1.1 [31]. Lesions judged to become visible as a result of treatment*, e.g.*, “new” sclerotic bone metastases, are not included in assessment of PD. Clinical progression is defined as clinical worsening of disease-related symptoms which may be supported by a significant and continuous rise in serum CA 19–9.

#### Overall survival

OS is defined in the ITT population as time from date of randomization to date of death of all causes.

#### Overall response rate

In patients with measurable disease at baseline, RECIST ver.1.1 will be used for evaluation of complete response (CR), partial response (PR), stable disease (SD) or PD. ORR will be calculated as the percentage of patients with CR and PR of all patients with measurable disease who received at least one treatment and were evaluated by at least one diagnostic CT scan. No centralized review of scans is planned.

#### Toxicity during treatment

All grades of toxicity are registered from the date of start of treatment to at least 28 days after the last dose or until the end of study visit. The cumulative worst toxicities ≥ CTC grade 3 in the PP population are calculated and compared for each randomization arm.

#### Quality of life

QoL will be assessed in the PP population in patients who have completed at least the baseline and one-follow-up questionnaire. The EORTC Core QoL questionnaire (EORTC QLQ-C30) is designed to measure cancer patients’ physical, psychological and social functions and has been validated for use in prospective clinical trials and is translated to Danish [32, 33]. According to the EORTC guidelines, a questionnaire will be analyzed if more than 50% of the items are completed. Otherwise, questionnaires will be considered as missing [34]. Missing single items are treated as missing. QoL scores collected will be linearly transformed to a scale of 0 to 100 [34]. Items will be grouped in health status scale (range 0–100, high is better), functional scales (range 0–100, high is better) and symptom scales (range 0–100, low is better). Each scale is summarized by its mean with standard deviation for the patients in the two treatment arms. The difference in mean at 8, 16, and 24 weeks is compared to the baseline mean within in each treatment arm using the Student’s t test [35].

#### Rate of hospitalizations during treatment

The rate, i.e., number (and mean) per patient per month, of hospital admissions in a stationary unit with overnight stay from the start of treatment to the date of end of treatment will be assessed for each randomization arm in the PP population. If the patient is readmitted for the same reason within 3 days *(e.g.*, after weekend leave), this is not counted as a separate admission. The reasons for admission are registered as toxicity due to treatment, symptoms due to PC, or other reasons.

### Exploratory endpoints

Patients who consent will, in addition to the clinical protocol, be included in the national Danish BIOmarkers in patients with PAncreatic Cancer (BIOPAC) protocol (NCT03311776) with blood samples for research taken at regular intervals [36, 37]. The blood samples will be handled and stored according to standard operating procedures described in the BIOPAC protocol [38], which also allows for tissue-based analyses on archived material.

#### Blood biomarkers

Markers of activation of the systemic inflammatory response including high neutrophil-to-lymphocyte ratio [39], elevated C-reactive protein (CRP), as well as the combination of CRP and albumin in the modified Glasgow Prognostic Score, have been associated with poor prognosis in PC [40, 41]. Other pro-inflammatory biomarkers include chitinase-3-like protein 1 (CHI3L1) (also known as YKL-40) and the cytokine interleukin-6 (IL-6) [42–44]. The combination of high CRP, YKL-40, and IL-6 levels has been shown to be associated with poor prognosis in patients with advanced PC [45]. In the present study we will assess the inflammatory response at baseline and during treatment and its impact on outcome.

Although no large-scale prospective studies have been published, liquid biopsies using blood samples as sources of tumor-derived genetic material may prove useful for prediction of prognosis and early assessment of treatment response in PC [46]. Among other candidate measures, a prior study suggested that promotor hypermethylation (ph) of secreted frizzled-related protein 1 (SFRP1) in plasma cell free DNA may be a prognostic marker in Gem-treated PC patients [47]. In the present study the prognostic and predictive value of ctDNA assessments will be investigated.

#### Tissue biomarkers

Resistance to chemotherapy, either primary or secondary, is ubiquitous in advanced PC [48]. One of several resistance mechanisms is through upregulation of ATP-binding cassette (ABC) proteins [49] These are transmembrane proteins located in several kinds of tissues including the pancreas that can pump molecules toward their gradient across plasma and intracellular membranes, reducing the concentration of intracellular chemotherapy [49]. High expression of ABC-B1 and ABC-G2 proteins has been correlated with resistance to taxanes in preclinical studies [50] and high expression levels of ABC-G2 were associated with poor prognosis in patients with resectable PC treated with adjuvant gemcitabine [51]. In formalin-fixed paraffin-embedded (FFPE) archival tissue we will investigate tissue expression levels of ABC protein subtypes. We hypothesize that levels are predictive for outcome of patients receiving GemNab, but not for Gem, and thereby may be useful for future stratification of patients to different types of chemotherapy.

#### Measures of frailty

In elderly and frail patients several issues may influence the patient’s well-being, such as comorbidity, polypharmacy, physical and psychological functioning and social status. These aspects are assessed in a comprehensive geriatric assessment (CGA), which is recommended by the International Society of Geriatric Oncology (SIOG) [52]. Several screening tools have been developed to help select the patients who may benefit from a CGA. G8 is an eight-item screening tool [53] covering several domains: nutrition (declining food intake, weight loss, Body Mass Index), comorbidities (polypharmacy), cognition/depression, and mobility, as well as age and self-rated health. The G8 has shown an ability to predict functional decline [54], to be associated with chemotherapy-related toxicity [55] and to be a prognostic marker [54, 56]. The modified G8 (mG8) is less investigated [57], however, an association with short- and long-term survival has been found [58]. It consists of 6 items covering nutritional status in terms of weight loss, polypharmacy, previous heart failure or coronary artery disease, cognition and mood, self-rated health and a simplified version of PS. Finally, the chair-stand-test is used to measure physical function and lower limb strength. It is a validated test with a low test–retest variability [59]. A slow chair-stand-test is associated with worsening activities of daily living (ADL) in older, community-dwelling adults and may be improved by multimodal exercise intervention [60]. The thresholds used for reporting the geriatric screening results are according to literature. The present study will include the above-mentioned measures of frailty, to assess whether scoring would enable a personalized allocation to different treatments or indicates a possibility for interventions.

### Statistical considerations

We are planning a randomized study to test the null hypothesis, that the mPFS in the control (Gem) and experimental arm (GemNab in 80% dose) is equal, opposed to the alternative hypothesis of being nonequal. The study will include 1 control per experimental subject. If the true mPFS in the control and experimental arm is 3 [7] and 5.5 [8] months, respectively, and the respective hazard functions in each group can be assumed to be constant and a log-rank test is used to test the hypothesis, we will need to include 50 subjects in each arm to be able to reject the null hypothesis with a power of 0.8 and a type I error of 0.05. For the power calculation, we used the procedure PROC POWER from SAS software version 9.4 (Copyright © 2016 SAS Institute Inc.).

PFS and OS will be estimated by Kaplan–Meier methods and compared with log-rank test. Patients who are alive will be censored at the last known time the patient is alive. PFS and OS will be summarized by mPFS and mOS along with the hazard ratios (HRs) and including 95% confidence interval (CI).

Patient demographics, baseline characteristics and AEs will be described using descriptive statistics. Continuous variables will be summarized with medians and ranges. Categorical variables will be summarized with frequencies and percentages (including 95% CI).

### Study status

The trial will be initiated June 2023. The plan is to recruit 100 patients in 18 months. It is estimated that each patient will receive treatment for an average of approximately 5 months and additional follow-up after the accrual interval will be 6 months. The entire study is thus projected to be concluded within 2 years.

## Discussion

The optimal treatment of frail patients with PC is understudied, which is counterintuitive to the fact that the majority are in poor PS and/or elderly. The design of this study was inspired by studies in vulnerable patients with colorectal cancer showing improved PFS and more manageable toxicity with reduced dose combination chemotherapy as compared to full dose single-drug treatment [61] as well as results of a post hoc analysis of GemNab in metastatic PC, suggesting improved survival for patients who had dose reductions or delay [14]. A comparison of two dose-reduced combination regimens is to be explored in another national randomized phase II trial in progress (NCT04233866), recently described by Dotan et al [62]. In this trial patients with PC in PS 0–2 with mild abnormalities in functional status and/or cognition, moderate comorbidities, or over age 80 are randomized to dose-reduced treatment with GemNab every other week or dose-reduced 5-FU plus liposomal irinotecan every other week. As we intend to compare results of standard dose Gem with reduced dose GemNab, the combined outcomes of these trials may define a future new standard of care.

Equally important to the lifes of very poor prognosis patients is how QoL is affected by treatment. In the Burris trial, patients treated with Gem had a greater clinical benefit score (derived from the measurement of pain, functional impairment and weight loss) as compared to 5-FU [5]. In the MPACT trial, QoL was not investigated, but quality-adjusted time without symptoms of disease progression or toxicity (Q-TWIST) was calculated in a later analysis [63]. Patients treated with GemNab had a significantly longer Q-TWIST (8.2 months) compared to those treated with Gem (6.5 months)[63]. In a prospective observational study, QoL was investigated in 600 patients with PC receiving GemNab in standard dose [64]. Three months after treatment start 61% of patients maintained their QoL score and the median time to deterioration was 4.7 months [64]. In contrast, in a phase II trial of 80 elderly patients receiving GemNab in standard dose, the median time to deterioration was only 1.6 months and 63% experienced grade 3–4 AEs, and it was concluded that GemNab did not confirm a QoL benefit in elderly [65]. The design of the present study enables, for the first time, a direct comparison of QoL of patients treated with either Gem or reduced dose GemNab.

In conclusion, single-drug gemcitabine has for more than thirty years been the main treatment option for vulnerable patients with non-resectable PC, but the impact on outcome is modest. If improved efficacy and sustained tolerability with reduced dose combination chemotherapy can be demonstrated, this could be changing future practice.

## Supplementary Information

**Additional file 1:** **Table S1**. Dose levels for Arm A. **Table S2**. Dose modifications for hematologic toxicity at start of each cycle or within a cycle for arm A. **Table S3**. Dose modifications for other toxicities for Arm A. **Table S4**. Dose levels for Arm B. **Table S5**. Dose modifications for hematologic toxicity at start of each cycle or within a cycle for Arm B. **Table S6**. Dose modifications for other toxicities for Arm B. **Table S7**. Summary of scheduled investigations.

## Data Availability

Not applicable.

## Abbreviations

PC: Pancreatic cancer
Gem: Gemcitabine
GemNab: Gemcitabine and nab-paclitaxel
DPCG: Danish pancreas cancer group
ECOG: Eastern cooperative oncology group
IL: Interleukin
FU: Fluorouracil
mOS: Median overall survival
KPS: Karnofsky performance status
PS: Performance status
AE: Adverse event
ESMO: European society of medical oncology
RCT: Randomized clinical trial
mPFS: Median progression-free survival
ORR: Overall response rate
QoL: Quality of life
ctDNA: Circulating tumor DNA
NCI: National cancer institute
CTC: Common terminology criteria
ITT: Intention-to-treat
PP: Per protocol
PD: Progressive disease
G-CSF: Granulocyte-colony stimulating factor
EORTC: European organization for research and treatment of cancer
QLQ: Quality of life questionnaire
CCI: Charlson comorbidity index
G8: Geriatric 8
mG8: Modified Geriatric 8
CA: Cancer antigen
CT: Computerized tomography
RECIST: Response evaluation criteria in solid tumours
CR: Complete response
PR: Partial response
SD: Stable disease
SAE: Serious adverse event
BIOPAC: BIOmarkers in patients with PAncreatic Cancer
CRP: C-reactive protein
CHI3L1: Chitinase-3-like protein 1
ph: Promotor hypermethylation
SFRP1: Secreted frizzled-related protein 1
ABC: ATP-binding cassette
CGA: Comprehensive geriatric assessment
SIOG: International society of geriatric oncology
ADL: Activities of daily living
HR: Hazard ratio
CI: Confidence interval
IHC: International council for harmonisation
GCP: Good clinical practice
GDPR: General data protection regulation
ICMJE: International committee of medical journal editors
Q-TWIST: Quality-adjusted time without symptoms of disease progression or toxicity
