# Supplementary material for: A randomized phase II study of full dose gemcitabine versus reduced dose gemcitabine and nab-paclitaxel in vulnerable patients with non-resectable pancreatic cancer (DPCG-01)
Source: BMC Cancer. 2023 Jun 16;23:552. doi: 10.1186/s12885-023-11035-6 (PMC10273702; doi:10.1186/s12885-023-11035-6)
Supplement: Supplementary file 1 — Additional file 1: Table S1. Dose levels for Arm A. Table S2. Dose modifications for hematologic toxicity at start of each cycle or within a cycle for arm A. Table S3. Dose modifications for other toxicities for Arm A. Table S4. Dose levels for Arm B. Table S5. Dose modifications for hematologic toxicity at start of each cycle or within a cycle for Arm B. Table S6. Dose modifications for other toxicities for Arm B. Table S7. Summary of scheduled investigations. [file 12885_2023_11035_MOESM1_ESM.docx]

**Supplementary table S1-7**

**Table S1: Dose levels for Arm A**

| **Dose level** | **Gemcitabine dose (mg/m^2^)*** |
| --- | --- |
| Start dose | 1000 |
| Dose reduction level - 1 | 800 |
| Dose reduction level - 2 | 600 |

* Day 1, 8 and 15 every 4 weeks.

**Table S2: Dose modifications for hematologic toxicity at start of each cycle or within a cycle for arm A**

| **Cycle day** | **Neutrophils (10**^9^**/L)** |  | **Platelets**  **(10^9^/L)** | **Gemcitabine dose** |
| --- | --- | --- | --- | --- |
| **Day 1** | | | | |
|  | $\geq$ 1.5 | AND | $\geq$ 100 | Treat on time |
|  | $<$ 1.5 | OR | $<$ 100 | Delay by 1-week intervals until recovery |
| **Day 8** | | | | |
|  | $\geq$ 1 | AND | $\geq$ 75 | Treat on time |
|  | $\geq$ 0.5 but $<$ 1 | OR |  | Decrease dose by 1 level* |
|  | $<$ 0.5 | OR | $<$ 50 | Hold |
| **Day 15: If Day 8 doses were given without modification** | | | | |
|  | $\geq$ 1 | AND | $\geq$ 75 | Treat on time |
|  | $\geq$ 0.5 but $<$ 1 | OR | ≥ 50 but < 75 | Decrease Day 8 dose by 1 level* |
|  | $<$ 0.5 | OR | $<$ 50 | Hold |
| **Day 15: If Day 8 doses were reduced** | | | | |
|  | $\geq$ 1 | AND | $\geq$ 75 | Same dose as Day 8 |
|  | $\geq$ 0.5 but $<$ 1 | OR | ≥ 50 but < 75 | Decrease Day 8 dose by 1 level* |
|  | $<$ 0.5 | OR | $<$ 50 | Hold |
| **Day 15: If Day 8 doses were interrupted** | | | | |
|  | $\geq$ 1 | AND | $\geq$ 75 | Decrease dose by 1 level* |
|  | $\geq$ 0.5 but $<$ 1 | OR | ≥ 50 but < 75 | Decrease dose by 2 levels^ |
|  | $<$ 0.5 | OR | $<$ 50 | Hold |

*Hold if dose is already decreased by 2 levels.
^Hold if dose is already decreased.
G-CSF is permitted in case of dose-limiting neutropenia. Patients experiencing drug related toxicities that require a delay $>$ 21 days will be discontinued from further treatment. If dose is held for 2 previous consecutive cycles, then decrease dose by 1 level throughout the rest of the treatment.

**Table S3: Dose modifications for other toxicities for Arm A**

| **Toxicity** | **Gemcitabine dose** |
| --- | --- |
| **Febrile neutropenia** | Hold until resolution of fever end returning of blood counts. Decrease dose by 1 level throughout the rest of the treatment^#^* |
| **Cutaneous toxicity** ≥ **grade 2** | Decrease dose by 1 level throughout the rest of the treatment*; if the patient continues to experience these reactions despite dose reduction, treatment should be discontinued |
| **Other toxicities ≥ grade 3**  (except nausea/vomiting and alopecia) | Hold until resolution to ≤ grade 1. Then decrease dose by 1 level* |
| **Symptomatic interstitial pneumonitis** | Treatment should be discontinued |

*Treatment should be discontinued if dose is already decreased by 2 levels.

^#^G-CSF should be considered.

**Table S4: Dose levels for Arm B**

| **Dose level** | **Nab-paclitaxel dose (mg/m^2^)*** | **Gemcitabine dose (mg/m^2^)*** |
| --- | --- | --- |
| Start dose | 100 | 800 |
| Dose reduction level -1 | 75 | 600 |

* Day 1, 8 and 15 every 4 weeks.

**Table S5: Dose modifications for hematologic toxicity at start of each cycle or within a cycle for Arm B**

| **Cycle Day** | **Neutrophils**  **(10^9^/L)** |  | **Platelets**  **(10^9^/L)** | **Nab-paclitaxel and gemcitabine dose** |
| --- | --- | --- | --- | --- |
| **Day 1** | | | | |
|  | ≥ 1.5 | AND | ≥ 100 | Treat on time |
|  | < 1.5 | OR | < 100 | Delay by 1-week intervals until recovery |
| **Day 8** | | | | |
|  | ≥ 1 | AND | ≥ 75 | Treat on time |
|  | ≥ 0.5 but < 1 | OR | ≥ 50 but < 75 | Decrease dose by 1 level* |
|  | < 0.5 | OR | < 50 | Hold |
| **Day 15: If Day 8 doses were given without modification** | | | | |
|  | ≥ 1 | AND | ≥ 75 | Treat on time |
|  | ≥ 0.5 but < 1 | OR | ≥ 50 but < 75 | Decrease dose by 1 level* |
|  | < 0.5 | OR | < 50 | Hold |
| **Day 15: If Day 8 doses were reduced** | | | | |
|  | ≥ 1 | AND | ≥ 75 | Same dose as Day 8 |
|  | < 1 | OR | < 75 | Hold |
| **Day 15: If Day 8 doses were interrupted** | | | | |
|  | ≥ 1 | AND | ≥ 75 | Decrease dose by 1 level* |
|  | < 1 | OR | < 75 | Hold |

*Hold if dose is already decreased.
G-CSF CSF is permitted in case of dose-limiting neutropenia. Patients experiencing drug related toxicities that require a delay > 21 days will be discontinued from further treatment.
If dose is held in 2 previous consecutive cycles, then decrease dose by 1 level throughout the rest of the treatment.

## **Table S6: Dose modifications for other toxicities for Arm B**

| **Toxicity** | **Nab-paclitaxel dose** | **Gemcitabine dose** |
| --- | --- | --- |
| **Febrile neutropenia** | Hold until resolution of fever end returning of blood counts. Decrease dose by 1 level throughout the rest of the treatment^#^* | |
| **Peripheral neuropathy  ≥ grade 3** | Hold until resolution to ≤ grade 1. Then decrease dose by 1 level throughout the rest of the treatment*^ | Treat on time |
| **Cutaneous toxicity**  **≥ grade 2** | Decrease dose by 1 level throughout the rest of the treatment*; If the patient continues to experience these reactions despite dose reduction, treatment should be discontinued | |
| **Other toxicities ≥ grade 3**  (except nausea/vomiting and alopecia) | Hold until resolution to ≤ Grade 1. Then decrease dose by 1 level* | |
| **Symptomatic interstitial pneumonitis** | Treatment should be discontinued | |

^#^G-CSF should be considered.

*Hold if dose is already decreased.

^Patients experiencing peripheral neuropathy that requires a delay for > 21 days will discontinue nab-paclitaxel, however, gemcitabine treatment can continue.
Patients experiencing other drug related toxicities than peripheral neuropathy that require a delay > 21 days will be discontinued from further treatment with both nab-paclitaxel and gemcitabine.

**Table S7: Summary of scheduled investigations**

|  | **Baseline** | **Prior to each treatment** | **Prior to day 1 in each cycle** | **Every 8 weeks of treatment** | **At time of progression** |
| --- | --- | --- | --- | --- | --- |
| Medical history, height | X^1^ |  |  |  |  |
| CCI^a^, G8 test^b^, mG8 test^c^ | X |  |  |  |  |
| Chair-stand-test^d^ | X |  |  | X |  |
| Weight and PS | X^1^ |  | X |  | X |
| Intercurrent hospitalizations |  |  | X |  | X |
| Physical examination, symptoms and toxicity | X^1^ |  | X |  | X |
| Hematology^e^ | X^1^ | X | X |  |  |
| Liver and renal chemistry^f^ | X^1^ |  | X |  |  |
| CRP | X^1^ |  |  |  |  |
| CA 19-9 | X^1^ |  | X^3^ | X |  |
| Optional blood samples for biomarkers | X |  | X^3^ | X | X |
| QoL questionnaire^g^ | X |  |  | X^4^ |  |
| CT scan | X^2^ |  |  | X |  |

1: Within 2 weeks before inclusion

2: Within 4 weeks before inclusion

3: Only prior to the second treatment course

4: Baseline and after 8, 16, and 24 weeks

1. CCI; Carlson Comorbidity Index based on 17 specific comorbid conditions. Each condition is assigned a weight from 1 to 6, based on the adjusted risk of mortality, and the sum of all weights results in a comorbidity score for the patient. The higher the score, the more likely the predicted outcome will result in mortality^1,2^. The CCI score includes two items for solid tumors: 1) Has the patient cancer? and 2) Has the patient a metastatic solid tumor? 1) will be answered with yes for all patients, 2) will be answered with yes, for patients with metastatic PC and no for patients with non-metastatic PC.
2. G8 test score ranges from 0-17. A score ≤ 14 is considered abnormal^3^.
3. Modified G8 test score ranges from 0-35, with more impairment following a higher score. Cut-off is ≥ 6, indicating potential frailty^3^.
4. Chair-stand-test is performed by the study nurse instructing the patient to raise from sitting position on a chair without arms to standing position as many times as possible during a 30 second time limit. The cut-off is depending on sex and age of the patient^4^.
5. Hematology includes hemoglobin, white blood cells, neutrophil, lymphocyte and platelet counts.
6. Liver and renal chemistry includes bilirubin, **aspartate** or alanine **a**mino**t**ransferase, and alkaline phosphatase, lactate dehydrogenase, albumen, creatinine and glomerular filtrating rate (measured or calculated).
7. EORTC Core QoL questionnaire (EORTC QLQ-C30) to be filled out by the patient before meeting the oncologist.

1. Charlson ME, Pompei P, Ales KL, MacKenzie CR. A new method of classifying prognostic comorbidity in longitudinal studies: development and validation. *J Chronic Dis*. 1987;40(5):373-383. doi:0021-9681/87

2. Charlson M, Szatrowski TP, Peterson J, Gold J, Gold J. Validation of a combined comorbidity index. *J Clin Epidemiol*. 1994;47(11):1245-1251. doi:10.1016/0895-4356(94)90129-5

3. Martinez-Tapia C, Canoui-Poitrine F, Bastuji-Garin S, et al. Optimizing the G8 Screening Tool for Older Patients With Cancer: Diagnostic Performance and Validation of a Six-Item Version. *Oncologist*. 2016;21(2):188-195. doi:10.1634/theoncologist.2015-0326

4. Rikli RE, Jones CJ. Functional Fitness Normative Scores for Community-Residing Older Adults, Ages 60-94. *J Aging Phys Act*. 1999;7(2):162-181. doi:10.1123/japa.7.2.162
